# The Therapeutic Treatment of Infected Tooth Root-Canals

**Published:** 1898-10

**Authors:** G. W. Warren


ARTICLE II.

### THE THERAPEUTIC TREATMENT OF INFECTED TOOTH ROOT-CANALS.

---

**Read before the Odontological Society of Pennsylvania, Oct. 8th, 1897.**

Mr. President and Fellows of the Odontological Society of Pennsylvania: The subject to which I shall ask your attention for a few minutes this evening, and which President Brubaker requested me to present, is not a new one, but is of much importance and full of interest to every practitioner of dentistry.

There have been many methods and remedies recommended for the treatment of putrescent tooth-canals; in fact, for nearly half a century this subject has laid claim to the attention of dentistry. To be more accurate, it was about forty-five years ago that it was first considered feasible to restore to usefulness, by therapeutic treatment, a tooth which had been unfortunate enough to have lost the vitality of its principal nourishing organ—the pulp. As far as I can learn, the pioneers in this opinion and treatment were the late Dr. Elisha Townsend and the venerable Dr. John Rich, of New York, now of Washington, D. C., who were soon followed by Atkinson, of New York, and Flagg, Truman and Pierce, of Philadelphia. In some respects the treatment to-day is precisely the same as recommended by these gentlemen at that time. First, they advised free access to the infected parts; secondly, cleanliness — that is, the free removal of the decomposed parts and the irrigation of the canals. These to-day are recognized as the two necessary preliminary steps. It is upon the following two that there has been such a diversity of opinion—that is, the remedies to be used as antiseptics and germicides, and the material best adapted for filling the root-canals after the treatment.

The first remedy claiming to possess the necessary properties for the successful treatment of such teeth, whether there was an alveolar abscess or not, was a saturated solution of iodine in creosote. This proved so unpleasant to handle and unsatisfactory in its results that it was soon discarded. Then followed carbolic acid and pure beechwood creosote. These have all had their advocates, have been experimented with, and by many discarded. Iodoform was introduced and used by many, having its advocates to-day. Also, bichloride of mercury and chloride of zinc have been employed to a considerable extent. One of the firmest adherents to the last two drugs was the late Dr. Abbott, of New York. He claimed, I believe, to use bichloride of mercury for putrescent canals and chloride of zinc in the treatment of teeth with alveolar abcesses, to the exclusion of all other remedies.

A more recent system is that introduced by Dr. Emil Schreier, of Vienna, known as the kalium-natrium (potassium and sodium) treatment. This preparation of potassium and sodium has found favor with many practitioners. It is clamed that by introducing it into a necrotic pulp or the putrescent contents of a root-canal, which consist largely of water, fats, and fatty acids, potassium and sodium hydroxides are formed, which, in combination with the fat of the pulp, form soap. This saponified material is readily removed and the canal filled Then came the recommendation of nitrate of silver. Nearly five years ago I received a communication from Dr. C. T. Stockwell, of Springfield, Mass., which was published in the International Dental Journal, recommending the use of this drug, especially in the treatment of those canals which we sometimes find, where it is very difficult or impossible to reach and thoroughly cleanse the extremity.

He said, in part, "With the rubber dam in place, I introduce and carry as far as possible into the canals a solution, fifty per cent. and upwards, of nitrate of silver, afterwards sealing in the chamber a pledget of cotton saturated with the same, and let it remain for a day or two. Then fill as successfully as possible with such material as circumstances indicate." My feeling in this direction was that the possibility of permently discoloring the teeth so treated being great, I should treat it in my practice as I did the introduction of copper amalgam into this country —let it entirely alone; leaving to the more zealous the experimentation along these lines. After waiting this length of time, I wrote to Dr. Stockwell a few weeks ago asking for results. His reply indorses my previously formed opinion of the drug, wherein he says, "I now seldom use it; it does the work, but I feel so sure of the teeth becoming darkened thereby that I use it only in those cases where other methods do not seem practical.

Among other preparations which have been brought prominently before us in recent years is sulphuric acid and sodium peroxide, and these have, in the hands of some practitioners, given considerable satisfaction. There are also other preparations which have been recommended, but I shall not occupy your time by enumerating them.

It is my belief that the relative value of drugs cannot be dissociated from certain medicinal properties, and that these properties are not only determined by scientific investigations through experiments in the test-tubes in our laboratories, but in addition to this, and of greater importance, I should say, is the experience derived from the every-day practice of careful and studious practitioners. We all know that certain drugs, when surrounded by certain conditions, will give us certain definite results, but let us change the conditions, as from the test tube to the root-canal surrounded by an inflammatory condition, and our results are often quite different. Now, I feel sure of my position in stating that it is the consensus of opinion with a larger number of teachers and experimenters that the agents we should employ in the treatment of putrescent root-canals should be (1) germicidal, (2) penetrating, and (3) non-irritating in their effect. And after a varied clinical experience I believe the essential oils more nearly meet these requirements than any other remedies we have at hand; notably, the oil of cinnamon, oil of eucalyptus, and oil of cloves. These oils have decided germicidal properties, are penetrating, and are mostly non-irritating to the tissue, and, in addition, their viscidity no doubt prevents, in a measure, the passage of bacteria.

I will briefly give you a report of two selected cases illustrating the line of treatmeut which I have indicated. About three years ago a lady called upon me for treatment, who, in the pursuance of her profession, is required to travel from city to city during the greater part of each year, thus requiring and receiving attention from several dentists. She reported that the first inferior molar on the right side had for a long while been the source of much annoyance. It had been treated by numerous dentists, would apparently return to the normal condition, but with every cold she contracted the alveolar inflammation would return. I inspected the tooth in question, and found in it a large amalgam filling; while on the border of the maxilla immediately below, and opposite the anterior root, was an inflammatory bunch, such as we have all seen, which seemed quite dense, and sore to pressure. I removed the filling from the tooth, and from the roots took a quantity of cotton, having a composite odor, in which I could detect that of creosote. I enlarged the canals slightly by means of the Gates Glidden drills, irrigated them thoroughly with peroxide of hydrogen and warm water, then introduced on a few fibres of asbestos a dressing of oil of cinnamon, closing the cavity with gutta-percha. The patient was visiting my office nearly every day for the week following, having a partial bridge denture inserted, which gave me an opportunity to watch the swelling disappear and the tissues return to a normal color and condition. At the close of her stay in this city she called, as I had requested, to have the tooth filled. I removed the dressing, dried the canals thoroughly with warm air, and closed the apical ends with a small poinc of gutta-percha; then filled the roots with oxychloride of zinc, and closed the crown cavity with an alloy filling and dismissed the case. Now we have the most interesting and important part of the matter. After an absence of two years the patient again called upon me for services, and reported that this tooth had not given her a moment's uneasiness during her abscence, and had been quite as useful as any other of the similar organs.

Case number two is the treatment of the left superior lateral incisor for a lady forty years of age. It so happened that I was inserting an artificial crown on either side of the tooth mentioned, and it was the opinion of the patient that the diseased tooth should be extracted and the space bridged. This tooth had for many months troubled the patient, being somewhat loosened, and there being a slight discharge of pus through a fistulous opening on the gum opposite the apical end of the root. It had been treated in Chicago and in this city, the last dentist filling the root with gold. After securing the patient's permission to treat the tooth, I removed the gold from the canal, washed thoroughly with peroxide of hydrogen, then with my hypodermic syringe I injected oil of cloves into the canal until it ran out through the fiistula on the gum; this was followed by a few fibres of asbestos saturated with oil of cloves being placed in the canal and the cavity closed with gutta-percha. After remaining about three weeks without any evidence of a recurrence of the discharge of pus, the dressing was removed, and a point of gutta-percha was passed into and through the end of the root until it made its appearance at the gum surface, and the cavity filled with the same material. The patient then left the city for the summer months. Upon her return in the autumn, I found that the tooth was entirely comfortable, quite firm, and that the tissues had settled down to a more normal condition, leaving the point of gutta-percha standing slightly above the surface; this point was then grasped with a pair of tweezers and twisted off at the end of the root. A permanent filling was then placed in the tooth, which has been a useful organ of mastication unto this time, there being no recurrence of the inflammatory condition.

In bringing these cases before you I do not present them as isolated ones, but as cases of frequent occurrence, illustrating, as I believe, the value of the essential oils in the therapeutic treatment of infected root canals.—G. W. Warren, in *International Dental Journal.*

